# Aspirin: a review of its neurobiological properties and therapeutic potential for mental illness

**DOI:** 10.1186/1741-7015-11-74

**Published:** 2013-03-18

**Authors:** Michael Berk, Olivia Dean, Hemmo Drexhage, John J McNeil, Steven Moylan, Adrienne O'Neil, Christopher G Davey, Livia Sanna, Michael Maes

**Affiliations:** 1 School of Medicine, Deakin University, 75 Pigdon's Road, Waurn Ponds, Geelong, Victoria, 3216, Australia; 2 Department of Psychiatry, University of Melbourne, Parkville, Victoria, 3052, Australia; 3 Orygen Youth Health Research Centre, 35 Poplar Road, Parkville, Victoria, 3052, Australia; 4 Florey Institute of Neuroscience and Mental Health, University of Melbourne, Parkville, Victoria, 3052, Australia; 5 Department of Immunology, Erasmus Medical Center, 3015 CE Rotterdam, The Netherlands; 6 School of Public Health and Preventive Medicine, Monash University, Melbourne; 7 Maes Clinics @ TRIA, Bangkok, Thailand

**Keywords:** aspirin, depression, schizophrenia, dementia, inflammation, cytokines, neuroprogression, treatment, COX

## Abstract

There is compelling evidence to support an aetiological role for inflammation, oxidative and nitrosative stress (O&NS), and mitochondrial dysfunction in the pathophysiology of major neuropsychiatric disorders, including depression, schizophrenia, bipolar disorder, and Alzheimer's disease (AD). These may represent new pathways for therapy. Aspirin is a non-steroidal anti-inflammatory drug that is an irreversible inhibitor of both cyclooxygenase (COX)-1 and COX-2, It stimulates endogenous production of anti-inflammatory regulatory 'braking signals', including lipoxins, which dampen the inflammatory response and reduce levels of inflammatory biomarkers, including C-reactive protein, tumor necrosis factor-α and interleukin (IL)--6, but not negative immunoregulatory cytokines, such as IL-4 and IL-10. Aspirin can reduce oxidative stress and protect against oxidative damage. Early evidence suggests there are beneficial effects of aspirin in preclinical and clinical studies in mood disorders and schizophrenia, and epidemiological data suggests that high-dose aspirin is associated with a reduced risk of AD. Aspirin, one of the oldest agents in medicine, is a potential new therapy for a range of neuropsychiatric disorders, and may provide proof-of-principle support for the role of inflammation and O&NS in the pathophysiology of this diverse group of disorders.

## Introduction

Historically, treatment options for common neuropsychiatric disorders, including depression, schizophrenia, and bipolar disorder, have focused on medications that modify the activity of monoamine neurotransmitter systems. Monoamines may play a large role in the pathophysiology of these disorders, but the monoaminergic theory of illness has failed to deliver novel agents beyond the limited treatment options currently available. There is now a clear body of recent evidence to support an etiologic role for other factors in the pathophysiology of depression, schizophrenia, and bipolar disorder, including oxidative and nitrosative stress (O&NS), mitochondrial dysfunction, and activation of the immune-inflammatory system.

The immune system is classically divided into the innate and adaptive arms. The innate immune arm is the first line of defense against pathogens, and provides a rapid response with limited specificity. Key players in the innate immune system are defense cells (such as neutrophils, monocytes, macrophages, natural killer cells, and mast cells), and soluble factors, of which acute-phase proteins, complement, and various inflammatory cytokines (such as interferon (IFN)-α, tumor necrosis factor (TNF)-α, interleukin (IL)-1β, IL-6, and IL-8) are examples. Monocytes are closely related to macrophages, and collectively these cells are often referred to as the 'mononuclear phagocyte system (MPS)'. The main descendants of these circulating monocytes are the macrophages, which occur in virtually all organs, and are present as microglia in the brain [1].

If the innate system fails to resolve the infection, the adaptive system will be triggered by the cells of the innate immune system. Dendritic cells pick up antigens at the site of infection, travel through the lymphatics to the lymph node, and present the collected antigen to cells of the adaptive immune system. The adaptive immune system is antigen-specific, provides a memory, and is typically activated a few days later than the innate system.

Inflammation, particularly the M1 macrophage response, is accompanied by increased levels of free radicals and O&NS, creating a state in which levels of available antioxidants are reduced. Activation of the immune-inflammatory and O&NS pathways and lowered levels of antioxidants are key phenomena in clinical depression (both unipolar and bipolar), autism, and schizophrenia [2-4]. Indeed, there is now strong evidence of the involvement of a progressive neuropathologic process in these conditions, with stage-related structural and neurocognitive changes well described for each. Incorporation of these wider factors into traditional monoamine neurotransmitter-system models has facilitated a more comprehensive model of disease, capable of explaining the observed process of neuroprogression. This understanding has facilitated the identification of new therapeutic targets and treatments that have the potential to interrupt the identified neurotoxic cascades [5-8]. The neuroprotective potential is one of the key promises of agents that target the components of the cascade.

One of the most commonly used pharmaceuticals in medicine, acetylsalicylic acid (aspirin), is an agent that may be capable of interrupting this neurotoxic cascade. Aspirin exerts its effects on the inflammatory cascades, irreversibly inhibiting cyclooxygenase (COX)--1, and modifying enzyme activity of COX-2, suppressing production of prostaglandins and thromboxanes [9]. These anti-inflammatory and anti-platelet mechanisms have been found to have positive effects on the risk of atherosclerosis, heart disease [10], stroke, and potentially, some cancers [11]. Given that similar disease mechanisms may underwrite the pathophysiology of major neuropsychiatric disorders, it is possible that aspirin, by interrupting neurotoxic cascades, could be a potential candidate for secondary prevention. In this review, we assess the evidence base supporting inflammatory and oxidative-stress disturbance in major neuropsychiatric disorders, and propose how aspirin may alter such pathways. Other reviews that have explored the use of somatic drugs for psychiatric diseases have placed a strong focus on COX-2 as the most important and powerful anti-inflammatory [12], but in this paper, we propose that COX-1 is in fact the key component in neurodegeneration and neuroinflammation, and thus a potential target for therapeutic intervention [12]. In addition, we identify some early indications of the preventative effect of aspirin on neuropsychiatric disorders.

## Inflammation and redox dysregulation in mood disorders

There is a large body of data showing that stress and depression are associated with both increased immune activation and impaired immune function [13]. To date, immune function in individuals with major depressive disorder (MDD) has been investigated by assessing levels of pro-inflammatory and acute-phase proteins [14]. Studies have shown that levels of interleukin (IL)-6 and C-reactive protein (CRP), and the immunologic complement component 4 (C4), but not C3, are significantly raised in patients with depression compared with controls [14]. Because increased levels of CRP predict the subsequent risk for *de novo *depression, these findings suggest that the documented immune findings are not merely epiphenomena of depression, but actually contribute to the genesis of the disorder [15]. A recent meta-analysis of cytokine studies in MDD showed that the cytokines IL-6 and TNF-α were increased [16]. There is evidence that the pro-inflammatory cytokine IL-1β and the pro-inflammatory chemokine CCL2 are increased in bipolar disorder [17,18].

Additional evidence for a central role of cytokines in depression derives from studies comprising healthy volunteers. For participants given infusions of endotoxin to induce the release of pro-inflammatory cytokines, there was a significant relationship between induced cytokine levels and emergence of mood-disorder symptoms [19]. Similarly, giving psychiatrically healthy individuals treatment with exogenous cytokines (IL-2, IFN-α and TNF-α) induced the classic behavioral and cognitive phenotype of a mood disorder, including depressed mood state, mania, increased stress reactions, cognitive impairment, and reduced motivation [20]. Recently, antidepressant treatment was shown to reduce levels of the inflammatory markers levels CRP and IL-6 in patients with MDD [21,22], while persistently raised levels of inflammatory markers were associated with lack of treatment response [23]. Indeed, such findings suggest an immunomodulatory role for antidepressant medication, and provide support for the use of immune interventions that directly address this aspect of the pathophysiology of depression.

There are equally extensive data on the relationship between oxidative stress (OS) and depression. Compared with healthy controls, individuals experiencing major depressive episodes (MDEs) have significantly increased levels of markers of oxidative damage, including lipid-peroxidation products [24-27], the oxidized DNA marker 8-hydroxy-2'-deoxyguanosine [28], and depleted omega-3 fatty acids (indicative of oxidative damage to erythrocyte membranes) [29]. Altered antioxidant levels are also seen in MDEs, including lower levels of serum vitamins C and E [30,31] and albumin [32], and altered levels of the antioxidative enzymes superoxide dismutase (SOD) [24-26,33], glutathione reductase (GR) and glutathione peroxidase (GSH-Px) [24]. A higher OS index, determined by the ratio of total plasma peroxide to total plasma antioxidant potential, is seen in drug-free patients with MDD [34]. Lastly, positive correlations have been found between depressive severity scores and OS index values [34], and between the severity of depression and the extent of changes in oxidative indices [24,26,28,31].

Normalization of oxidative parameters with the resolution of depression has been reported in two studies, in which it was found that baseline measures of antioxidant enzymes and lipid peroxidation were increased and vitamin C levels decreased in patients with MDE compared with controls [24,25]. Antidepressant treatment improved depressive symptoms, and was also associated with significant amelioration of oxidative parameters, corroborating findings from other studies [33]. Further supporting this, preclinical studies have shown that established antidepressant agents display antioxidant properties [35-38]. The evidence of a pathophysiological role of OS in MDD provides preliminary support for the use of antioxidant mechanisms as an additional therapeutic pathway.

## Inflammation and redox dysregulation in schizophrenia

In schizophrenia, as in depression, there is long-standing evidence to suggest a major role for OS and, to a lesser extent, inflammation; reduced levels of antioxidants, such as glutathione, have been reported as early as 1934 [39]. Since these early findings, there has been a plethora of studies indicating altered levels of antioxidants, including the major antioxidants SOD, GSH-Px, and catalase, in addition to glutathione [40]. Lipid peroxidation is evidenced by the increased levels of malondialdehyde (MDA) and thiobarbituric acid that are seen in patients with schizophrenia. In addition, protein carbonylation and DNA damage is increased, leading to activation of apoptotic pathways, further disrupting normal brain function. Moreover, genetic polymorphisms have been identified that further implicate oxidative factors in the pathophysiology of schizophrenia. Polymorphisms in the glutamate-cysteine ligase gene, whose protein product is responsible for glutathione synthesis, occurs in bipolar disorder [41] and schizophrenia [42], but not in depression [43]. In a similar manner to its relationship with depression, positive correlations exist between levels of OS and symptom severity [40].

Although there is a substantial body of literature regarding maternal and prenatal inflammation and their role in schizophrenia, it has only been comparatively recently that the roles of inflammation in schizophrenia have been intensively investigated. Infectious agents including *Toxoplasma gondii, Chlamydia*, bornavirus cytomegalovirus, and influenza seem to increase the risk for schizophrenia, but it is possible that the immune response itself rather than the infection mediates this effect [44]. Raised maternal levels of the pro-inflammatory cytokine IL-8 during pregnancy are associated with an increased risk for schizophrenia in the offspring, independent of the cause of inflammation [45]. The glutamatergic and dopaminergic systems, known to be dysregulated in schizophrenia, have a modulating effect on the immune system and on tryptophan-kynurenine metabolism, both of which are involved in the pathophysiology of schizophrenia [46]. Markers of inflammatory processes have been found in post-mortem studies of brains from patients with schizophrenia [47].

Pro-inflammatory changes have been described in schizophrenia; a recent meta-analyses of cytokine studies in schizophrenia showed that cytokines such as IL-12, TNF-α, IFN-γ and soluble CD25 are raised in and are trait markers of schizophrenia [48], whereas IL-1β, IL-6 and TGF-β are state markers of acute schizophrenia [48]. Serum levels of the monocyte/macrophage cytokines, chemokines and adipokines CCL2, CCL4, TNF-α, IL-1β, IL-6, leptin, adiponectin, pentraxin-related protein (PTX3), plasminogen activator inhibitor (PAI)-1, osteoprotegerin (OPG) and intercellular adhesion molecule (ICAM)-1 were measured in individuals with chronic schizophrenia and compared with levels in healthy controls (n = 138). Levels of all measured immune compounds except PAI-1 and OPG were increased in the patients with schizophrenia. Multivariate analysis showed that these increases were linked to gender (ICAM-1, leptin, TNF-α and adiponectin), a higher body mass index (leptin, adiponectin), presence of hyperglycemia or diabetes (CCL4 and OPG), reduced high-density lipoprotein cholesterol or increased triglyceride (adiponectin and PTX3) levels, or presence of metabolic syndrome (CCL2, leptin, and adiponectin). IL-1β and IL-6 were the only immune compounds found to be increased in the serum of patients not affected by any of these aforementioned factors. Although many of the immune compounds were found to be linked to (components of) metabolic syndrome, the most dominant linkage was found with schizophrenia itself, confirming earlier reports of increased monocyte/macrophage activation being a key component for understanding the pathogenesis of schizophrenia, consistent with the meta-analysis showing that IL-1β and IL-6 are trait markers of schizophrenia. Interestingly, previous work has shown that serum IL-6 levels seem to correlate with a poorer prognosis [49]. Positron emission tomography (PET) imaging studies using the ligand PK11195, a marker of microglial activation, have also suggested an inflammatory process in schizophrenia [50].

## Inflammation and redox dysregulation in Alzheimer's disease

Alzheimer's disease (AD) is the most common cause of dementia in the elderly population, with over 33 million people affected worldwide [51]. AD is characterized by two major neuropathologic changes: deposition of intracellular neurofibrillary tangles composed of tau protein, and extracellular β-amyloid plaques [52]. The disease is also characterized by activation of astrocytes and microglia, which leads to production of a range of pro-inflammatory substances, including cytokines, chemokines, thromboxanes, and reactive oxygen species (ROS) [53], which over time may lead to cellular damage. It is suggested that deposition of β-amyloid plaques and tau protein (as neurofibrillary tangles) induces a chronic inflammatory state that is derived primarily from efforts to clear this debris [52]. During this chronic inflammatory state, substances released by astrocytes and microglia, such as ROS, nitric oxide (NO), and proteolytic enzymes, can not only cause direct toxic effects to the surrounding neuronal architecture, but also enhance further production of beta-amyloid, potentially contributing to disease progression. This process seems to involve both an upregulation in β-amyloid-42 production, and decreased production of soluble amyloid precursor protein, which potentially exerts neuroprotective effects [54]

Similar to psychiatric disorders, the interaction between inflammatory and OS responses appears to be important in AD. A reciprocal relationship seems to exist between beta-amyloid and OS, with both augmenting production of the other [55,56]. In addition, patient's with AD appear to exhibit lower levels of antioxidant substances and enzymes early in the disease course [57], which, when coupled with the declining function of antioxidant defenses in key brain regions with advancing age [58], may predispose those with AD to OS mediated damage.

## The association between psychiatric disease, dementia, and auto-immunity

To further support a role for therapeutic agents targeting inflammation in psychiatry, there is a large body of evidence linking autoimmune disease to psychiatric disorders. For example, clinical depression is associated with diverse autoimmune disorders, including diabetes type 1 and 2, inflammatory bowel disease, psoriasis, rheumatoid arthritis, atherosclerosis, lupus erythematosus, and multiple sclerosis (MS) [59]. Patients with clinical depression have a high degree of auto-immunity directed against a number of different selfepitopes, including serotonin and phospholipids (for example, cardiolipin and antinuclear factor) [60]. Recently, a new type of autoimmune response has been described, which is an autoimmune response secondary to O&NS damage [61-63]. Thus, it is possible that increased O&NS levels may damage endogenous molecules, such as fatty acids and proteins, thereby changing their structure. As a consequence, the O&NS-modified self determinants may be rendered immunogenic, and an autoimmune response is then directed against the modified epitopes (neo-epitopes) [61]. For example, clinical depression is accompanied by IgG-mediated immune responses directed against oxidized low-density lipoprotein. Moreover, there is an association between this kind of autoimmune response and progression (or staging) of depression. Consequently, some of these autoimmune responses are significantly higher in depressed individuals with chronic depression (duration of >2 years) compared with patients who are depressed but do not have chronic depression [60]. These findings suggest that O&NS damage, the consequent formation of neo-epitopes, an enhancement of the natural autoimmune response, and even a transition to pathological damaging auto-immunity increase the risk of neuroprogression and of chronic depression.

For schizophrenia, there is a parallel evidence base for an association with auto-immunity. In a large Danish national study, individuals with schizophrenia had a 45% higher chance of developing an organ-specific autoimmune disease, including thyroid autoimmune disease [64]. Moreover, these autoimmune diseases were also more prevalent in the parents of these individuals with schizophrenia. These findings not only imply a genetic component, with family members having a higher chance of developing such autoimmune disease, but indicate a putative shared immune pathogenesis of psychiatric disorder and organ-specific auto-immunity. Although organ-specific autoimmune diseases have been reported to have a higher prevalence in patients with schizophrenia, there are also numerous reports of a negative co-occurrence of schizophrenia and rheumatoid arthritis, which is a systemic autoimmune disorder, and the underlying immune mechanism is as yet unknown [65]. A number of autoantibodies are significantly higher in patients with schizophrenia compared with controls, especially those related to autoimmune responses directed against neuronal targets [66-68].

Evidence from the literature on bipolar disorder also supports the role of a genetic component; patients with bipolar disorder and their relatives have been shown to be more prone to develop thyroid auto-immunity, and this association is not attributable to the use of lithium or to the severity of psychiatric symptoms [69-71]. Moreover, in addition to a higher prevalence of thyroid autoantibodies, patients with bipolar disorder have a higher prevalence of organ-specific autoantibodies, including autoantibodies to hydrogen/potassium ATP and glutamic acid decarboxylase-65 [72]. The aforementioned Danish national study [64] confirmed these findings by showing an association of bipolar disorder with a family history of pernicious anemia, and with presence of Guillain-Barré syndrome, inflammatory bowel disease, and autoimmune hepatitis in individual patients.

Collectively, these findings imply shared immune pathogenic factors for mood disorders, schizophrenia, and organ-specific autoimmune diseases. One of these shared factors is thought to be an intrinsically high activation set-point for the MPS. It is thought that the high activation set-point of these cells of the MPS can be down-regulated by aspirin.

## Activated circulating monocytes in psychiatric disorders

There is emerging evidence that the MPS is activated in patients with psychiatric disease. Early reports show that the number of circulating monocytes is aberrant in patients with schizophrenia. Rothermundt *et al*., reported a slight increase in the mean absolute and relative monocyte counts [73], and others have supported these observations, showing the presence of monocytosis and a higher number of CD14+ cells in non-medicated patients with schizophrenia and in children with psychosis [74,75]. In contrast to schizophrenia, higher numbers of CD14+ monocytes could not be found in bipolar disorder [17,76], yet gene-expression studies identified activation of the circulating cells. Two gene-expression profiling studies [17,18] have been carried out on purified monocytes taken from patients with psychiatric illness (56 with bipolar disorder and 27 with schizophrenia) and matched healthy controls. In sum, aberrant expression of 34 genes was detected, which mutually correlated and formed an immune-activation monocyte gene-expression signature, consisting of pro-inflammatory cytokines and compounds (for example, IL-1β, IL-6 and COX2), inflammatory regulator molecules (for example, NAB-2), various chemokines (for example, chemokine (C-C motif) ligand (CCL)2), adhesion/motility factors (for example, cell division control protein 42 homolog; CDC42), and inflammatory transcription factors (for example, early growth response protein (EGR)3). The overexpression of monocyte activation genes was particularly evident in active cases, that is, in patients with mania, active melancholic depression, or active psychosis [17,18].

## Activated microglia in psychiatric disorders

Although histomorphologic signs of an abnormal inflammatory activation of microglia have been found in post-mortem studies on patients with major psychiatric disorders, these studies are limited and controversial.

A post-mortem study on the brains of patients with schizophrenia who had committed suicide during an episode of acute psychosis found an increased density of microglia in the tissue [77]. Three other studies reported increased microglial activation in patients with schizophrenia [78-80], whereas another three did not find an activation state of microglia [81-83]. A drawback of some of these post-mortem studies is that they were performed on old to very old individuals, and might not reflect the pathophysiology of acute exacerbations of psychiatric illness.

To date, only two histological studies have analyzed patients with affective disorders. A qualitative study of human leukocyte antigen (HLA)-DR showed increased expression of this surface marker on the microglia of the hippocampus and prefrontal cortex of patients with depression [78].

More recently, increases in the expression of quinolinic acid (QUIN) have been identified in ramified microglia in sub-regions of the anterior cingulate cortex of patients with severe depression [84]. The production of microglial QUIN from tryptophan was increased in the subgenual anterior cingulate cortex and anterior midcingulate cortex, but not in the pregenual anterior cingulate cortex of patients with major depression. A similar trend was seen for bipolar disorder. QUIN is a breakdown product of the tryptophan pathway, which is further broken down to NAD+, and is primarily produced by activated microglial cells. A key enzyme for the production of QUIN in the microglia is indoleamine 2,3-dioxygenase (IDO), which IDO is activated by pro-inflammatory cytokines including IFN-γ, IL-1, IL-6, and TNF-α.

In addition to post-mortem studies, techniques using PET have been used to study microglia activation in patients in real time. A PET tracer. [11C]-PK11195, binds to the mitochondrial translocator protein, whose expression is increased in activated microglia [85]. This technique has already been used successfully in several patient and animal studies of neuropsychiatric disorders [86], which show that immune-activation ('inflammatory') lesions occur in brain regions that are related to the specific disease process. For example, in schizophrenia, microglial activation is found in the hippocampal area, where functions such as immediate memory and sensory-emotional integration are impaired. Interestingly, these focal changes are found only in patients with acute psychosis, in whom cognitive impairment is most prominent [87], and not in patients who have recovered from psychosis [50], who show a global brain inflammatory effect.

Using PET, microglia activation has also been found in brain disorders such as AD, HIV-associated dementia, Parkinson's disease (PD), multisystem atrophy, MS, herpes encephalitis, traumatic brain injury, and stroke [88,89]. In addition, microglial activation is also found in peripheral disorders such as hepatitis, thiamine deficiency, and hepatic encephalopathy [90-92], and even after sleep loss [93].

An abnormal inflammatory activation of microglia can be detrimental for neurogenesis and synaptogenesis through lack of provision of neuronal growth factors or through production of neurotoxic factors and cytokines [94]. Mouse mutants in which microglia are in an activated state during prenatal development (CD200KO and DAP12KO mice) have altered levels of glutamate receptors, resulting in impaired long-term potentiation and hippocampal transmission [95-97]. In favor of a direct detrimental action of inflammatory cytokines on neuronal development, *in vitro *work has shown that cytokines such as IL-6, IL-18, and TNF-α can affect neuronal proliferation, survival, and aspects of differentiation such as neurite outgrowth and gene-expression patterns [98,99].

## Working mechanisms of psychotropic medications

For unipolar and bipolar depression, it is understood that the working mechanisms of classic antidepressants and new putative antidepressants are at least partly explained by their effects on various immune-related pathways, including: 1) reducing inflammatory responses characterized by increased levels of TNF-α, IL-1 and IL-6; 2) attenuating cell-mediated immune activation and T-helper (Th)1 and Th17 responses while increasing responses of regulatory T cells; 3) reducing O&NS processes and increasing the total antioxidative capacity; 4) protecting against damage to mitochondria and mitochondrial DNA; and 5) attenuating neuroprogressive processes [100]. The same immunoinflammatory, O&NS and neuroprogressive pathways play a role in schizophrenia [101]. Therefore, it has been suggested that targeting the aforementioned pathways in conjunction with dopamine receptor 2 and 5-hydroxytryptamine receptor 2 may be a treatment approach for schizophrenia [101].

## Working mechanisms of aspirin

Aspirin is a non-steroidal anti-inflammatory drug (NSAID), and an irreversible inhibitor of both COX-1 and COX-2. It is more potent in its inhibition of COX-1 than COX-2, and targeting COX-2 alone may be a less viable therapeutic approach in neuropsychiatric disorders such as depression [102]. COX-2 inhibitors may theoretically cause neuroinflammatory reactions, and potentially might augment the Th1 predominance, increase O&NS levels and O&NS-induced damage, decrease antioxidant defenses, and even aggravate neuroprogression [102]. In addition, COX-2 inhibition may interfere with the resolution of inflammation [103]. Thus, COX-2 inhibition decreases the production of prostaglandin E2 (PGE2), which drives the negative immunoregulatory effects on ongoing inflammatory responses. In autoimmune arthritis, for example, PGE2 is part of a negative-feedback mechanism that attenuates the chronic inflammatory response [103]. Therefore, in order to understand the clinical efficacy of aspirin in neuropsychiatric disorders such as depression and schizophrenia, it is more important to consider how its inhibition of COX-1 affects the five aforementioned pathways. This is supported by data suggesting lower response rates to antidepressants in people receiving NSAIDs [104], but is at odds with some recent studies suggesting a benefit for celecoxib, a COX-2 inhibitor, in several disorders including autism and depression [105,106]. In the following sections, we will discuss the effects of aspirin on these pathways.

## Aspirin and the suppression of activated cells of the mononuclear phagocyte system

It is relevant to consider the inflammatory reaction as a biphasic process, with an initial phase of induction and a second phase of resolution. During the initial phase of inflammation, eicosanoids including prostaglandins and leukotrienes play an important role as local mediators in the development of an inflammatory condition, evoking potent chemotactic responses from leukocytes, the activation of which is coupled to the production of pro-inflammatory cytokines at sites of inflammation [107]. The second stage of resolution is coupled to the biosynthesis of a new genus of lipid mediators that actively limit inflammation and promote resolution. This new genus of pro-resolving mediators includes lipoxins (LXs) and their aspirin-triggered carbon-15 epimers, as well as the recently discovered resolvins and protectins, which are derived from omega-3 fatty acids. An excellent review [108] on these new mediators has been published recently, and we give a summary below of the main principles described in this review.

LXs and aspirin-triggered LXs (ATLs) are considered to act as 'braking signals' in inflammation, dampening the inflammatory response. Aspirin triggers the generation of epimeric forms of LXs. Cells that express COX-2 (including activated monocytes, macrophages, and microglia) produce ATLs in response to the actions of aspirin, which triggers the endogenous formation of carbon-15 epimeric LXs. In particular, in a cytokine-primed milieu, acetylation of COX-2 by aspirin switches the catalytic activity of the enzyme to an R-lipoxygenase with the formation of 15R-hydroxyeicosatetraenoic acid, which is rapidly converted by 5-lipoxygenase to 15-epimeric-LXA4 or 15-epimeric-LXB4.

Administration of low doses of aspirin to healthy subjects significantly increases plasma levels of ATLs, with concomitant inhibition of thromboxane biosynthesis, suggesting that ATLs may account for some of the beneficial effects of aspirin that are not strictly related to its anti-thrombotic actions.

The role for ATLs as anti-inflammatory molecules is well defined, with their bioactions involving the inhibition of neutrophil and eosinophil recruitment and activation. In addition, LXs and ATLs have been proposed to directly stimulate expression of genes (such as NAB1) involved in endogenous anti-inflammation and resolution, and to regulate NF-κB activation.

The actions of LXs and ATL are not limited to counter-regulating the evolution of inflammation, as they also promote resolution at different levels. LXs stimulate monocyte chemotaxis and adherence, without causing degranulation or release of ROS, suggesting that the actions of LXs are related to the recruitment of monocytes to sites of injury. These monocyte activities may be host-protective. in view of the important role of these cells in wound healing and resolution at inflammatory sites. Indeed, LXs and ATLs stimulate the *in vitro *clearance of apoptotic cells by human monocyte-derived macrophages in a non-phlogistic manner. In addition to promoting resolution by non-phlogistic phagocytosis of apoptotic cells, LX can act to reprogram cytokine-primed macrophages from a classic pro-inflammatory phenotype to an alternatively activated phenotype.

A range of doses of aspirin (100 to 300 mg/day) reduced the plasma levels of inflammatory biomarkers such as CRP, IL-6 and TNF-α in patients with cardiovascular metabolic syndrome [109]. Aspirin was shown to reduce the levels of inflammatory cytokines, such as TNF-α and IL-8, but not those of negative immunoregulatory cytokines, such as IL-4 and IL-10 [110]. In the same study, there did not seem to be any effect of aspirin on IL-1β, and the suppressant effects of aspirin on IL-6 did not reach significance. Aspirin also reduced the production of TNF-α in rats with streptozotocin-induced type 2 diabetes [111]. Pretreatment with aspirin in various cell types, including keloid fibroblasts and HeLa cells, significantly reduced IL-1 and TNF-α-induced stimulation of nuclear factor (NF-)-κB [112,113]. In patients with chronic stable angina, treatment with aspirin for 6 weeks reduced serum levels of CRP and IL-6 [114]. Another study examined the effects of 2 weeks of treatment with oral aspirin (325 mg/day) on the *ex vivo *stimulated production of IL-1β, IL-6, and TNF-α by peripheral blood mononuclear cells from normal controls [115]. The authors found that oral aspirin may increase the production of cytokine-induced, but not lipopolysaccharide (LPS)-induced TNF-α and IL-1β production, suggesting that short-term treatment with aspirin might augment cytokine-induced cytokine production in normal controls. Taken together, these results provide some evidence that administration of aspirin to patients with inflammatory conditions may suppress the production of TNF-α, IL-1β and maybe IL-6, and of acute-phase proteins such as CRP. Aspirin also seemed to reduce Th17 responses in mouse models of LPS-induced lung inflammation [116].

There is also evidence that mRNA expression of COX-2 is increased in individuals with recurrent depression [117]. Likewise, a significant association was found between recurrent depression and a single-nucleotide polymorphism of the COX gene G-765C [118]. Nevertheless, as discussed above, these findings question the use of selective COX-2 inhibitors in clinical depression [102]. COX-2 has many effects, both negative and positive, on the central nervous system, meaning that the outcome of COX-2 inhibition in neuroinflammation is very difficult to predict [119]. For example, COX-2 has neuroprotective and anti-inflammatory effects, and may play a role in the integrity of the blood-brain barrier, synaptic transmission, and long-term potentiation [102,119-121]. These new data augment the view that COX-2 may not be the best target to inhibit neuroinflammation, and therefore targeting COX-1 could be a much better strategy to block neuroinflammation [119,120]. There are now data indicating that expression of COX-1 is enhanced in neuroinflammatory disorders, including models of PD, and that COX-1 inhibition improves survival [122]. Taken together, these new data show that COX-1, rather than COX-2, is a key component in neurodegeneration and neuroinflammation [123]. Thus, the working spectrum of aspirin (targeting preferentially COX-1 rather than COX-2) compared with that of selective COX-2 inhibitors may be the reason why aspirin is an asset in the treatment of neuropsychiatric conditions associated with neuroinflammation and neuroprogression.

It is thus tempting to speculate that aspirin is capable of deactivating and reprogramming the activated monocytes, macrophages, and T cells and the neuroinflammatory responses in neuropsychiatric disorders.

## Aspirin and the suppression of O&NS pathways

There is a vast literature on the anti-inflammatory effects of asprin. Since the discovery by Vane in 1971 of the inhibitory effect of on the COX system, multiple studies have focused on the effect of aspirin on systemic peripheral O&NS processes [124]. For example, aspirin blocks the IL-1β-induced production of NO and inducible NO synthase (iNOS) [125]. In cultured smooth-muscle cells, administration of aspirin significantly reduced the expression of iNOS [126], while in rat cardiac fibroblasts, aspirin suppressed iNOS induction [127]. By contrast, other authors found that aspirin and sodium salicylate actually increased iNOS and NO production in smooth-muscle cells [128]. The potential role for aspirin in modulating levels of O&NS pathways in the brain has received less attention. However, a number of animal studies support the contention that aspirin can act to reduce OS and prevent against oxidative damage. For example, administration of aspirin prior to acute stress exposure prevented increases in iNOS expression, TNF-α, and markers of lipid peroxidation (such as MDA) and OS [129]. Tables 1 and 2 provide a summary of the key evidence from observational and randomized controlled trials over the past 40 years, investigating the relationship between aspirin and mental disorders.

**Table 1:** Summary of key observational studies investigating the association between aspirin use and mental illness.

Author, year; study	Hypotheses	Study design	Agent and dosage	Sample	Psychiatric measure	Presentation of results	Key finding
Sturmer, 1996; EBSHP	ASA use affected decline of cognitive function	Cohort study	ASA: <1, 1 to 2 and, >2 tablets/day ASA effect hypothesized to be more dependent on frequency than on dose, mean daily dose was not used	3,631 NII >65 years old; 2,773 at 3-year follow-up, and 2,023 at 6-year follow-up	SPMSQ, EBMT	OR	No significant effect seen. Modest benefit of ASA, especially with intermittent use, on decline of cognitive function

Henderson, 1997	ASA prevented dementia or cognitive impairment	Community survey. Two-wave: cross-sectional at 2 wave; longitudinal from 1to 2 wave	ASA yes/no (unknown dose)	1,045 participants 70 years old at baseline; 588 people with cognitive assessment at both waves	CIE = MMSE, SLMT, NART	Mean and SD	Cross-sectional: no significant difference in cognitive tests. Longitudinal: no differences in cognitive decline or incidence of dementia

Stewart, 1997; BLSA	Reduced risk of AD in ASA users	Longitudinal	ASA yes/no; NSAIDs yes/no; acetaminophen yes/no	1,686 participants in baseline study	BIMCT, MMES, Immediate and Delayed Cued Recall, BNT, CVF, TMT A and B, Clock Drawing and other constructions, CESDS, PFAQ, NART	RR	Non-significant AD risk ratio for ASA users. Duration of ASA use and the risk of AD were not significantly associated

Peacock, 1999; ARIC study	Association of regular use of NSAIDs or ASA with cognitive function	Cross-sectional cohort study	NSAIDs yes/no; ASA yes/no	13,153 participants, 48 to 67 years old	Delayed word recall test, WAIS-R digit symbol subtest, WFT	Mean	Weak negative association (<0.1) between current ASA and word fluency & recall. No association of lifetime ASA and cognitive index scores. ASA treatment for 8 years weakly associated with better word fluency

Landi, 2003	Relationship between NSAIDs and AD	Cross-sectional	ASA yes/no; NSAIDs yes/no	2,708 participants admitted to home care programs	CPS	Mean and SD	NSAID users had a nearly 50% lower risk of cognitive impairment. For subjects using ASA, the risk estimated was similar; 67% decreased risk of cognitive impairment associated with non-ASA NSAID use

Nilsson, 2003; OCTO-Twin	ASA protective for AD	Cross-sectional and longitudinal	Low-dose ASA (75 mg/day + 3500 mg/week) 250, 500 mg	702 participants >80 years old, 91 with dementia at baseline; 88 developed dementia during observational period; 315 people at follow-up	DSM-III-R criteria for dementia, NINCDS/ARDRA criteria for AD, NINDS/AIREN criteria for vascular dementia, MMSE	β	At 9-year follow-up, significant association between ASA and lower frequency of AD and all dementia; significantly more likely that intact cognitive function was maintained in those taking ASA. Use of low-dose ASA alone did not affect the risk ratio

Cornelius, 2004; Kungsholmen Project	Association between ASA and NSAIDs with AD and overall dementia, and influence of apoE ε4	Longitudinal cohort study	ASA 75 to 500 mg/day yes/no; NSAIDs yes/no. Differences in dosages not considered	1,301 subjects >75 years old dementia-free at baseline; 987 at 1*follow-up (314 died); 650 at 2*follow-up (281 died)	DSM III-R criteria for dementia, Hachinski scale for differential diagnoses AD versus VaD versus Mixed dementia, MMSE, neurological and psychiatric examinations, neuropsychological assessment	Incidence	6-year increased AD risk in the ASA/apoE ε4 negative group

Jonker, 2004; LASA	Protective effect of ASA on cognitive decline in older people	Community-based study; ?case-control	NSAIDs yes/no; ASA <100 mg yes/no	612 participants, 62 to 85 years old	MMSE, AVLT, coding task	OR	3-year follow-up of decline in episodic memory (immediate recall) for ASA users was reduced by more than three times. Effect of ASA was significant only in >75-year-olds

Shepherd, 2004; SOP Study	ASA protective against AD		ASA yes/no	151 NII, >75 years old, dementia-free	MMSE, Logical Memory and Similarities subtest from WAIS-R, BNT, visuo-perceptual abilities from the JLOT, COWAT	Mean and SD	ASA use was a significant positive predictor of performance on the Logical Memory test

Arvanitakis, 2008; ROS	Relation between NSAIDs/AD, change in cognition, and AD pathology	Longitudinal	ASA yes/no; non-ASA NSAIDs yes/no	1,019 Catholic clergy, mean age 75 years old, dementia-free	As reported previously [12]	HR	At 1 year-no apparent relation of ASA to incident AD, change in cognition, or AD pathology

Szekely, 2008; CHCS	Association between NSAIDs, ASA, and acetaminophen with dementia and AD	Prospective	ASA yes/no; NSAIDs yes/no; acetaminophen yes/no. No dosage reported	3229 participants >65 years old, dementia-free 1228 ASA users	NINCDS/ARDRA criteria for AD, ADDTC criteria for VaD, Mixed dementia diagnosis, 3MSE, MRI	OR	At 10 years, risk of AD, VaD, and all-cause dementia was not associated with use of ASA

Almeida, 2010	ASA decreased prevalence of depression and cognitive impairment	Retrospective	Not reported	5,556 men 69 to 87 years old	GDS, MMSE	OR	ASA not associated with lower OR of depression or cognitive impairment in >75-year-old men. Discontinuation of ASA between the two assessments related to greater OR of depression than non-users

Pasco, 2010; GOS	ASA reduced the risk for depression; ASA + statin reduced risk of *de novo *depression	Case-control study, retrospective cohort analysis	ASA yes/no	386 women >50 - years old. 1* MDD >50 years old versus no MDD. No prior MDD, followed up from baseline or time of exposure to ASA, until 1* MDD or 10-year follow-up	SCID I RV-NP	OR and HR	OR for MDD in the ASA group was 0.18, *P *= 0.1 The prevalence of exposure to statins + ASA was lower for women with MDD; OR for MDD was 0.15 (95% CI 0.03 to 0.65, *P *= 0.01). Exposure to statins + ASA was associated with a reduced risk for MDD

Waldstein, 2010; BLSA	Relation between ASA and NSAIDs and age-related change in cognitive functions	Cross-sectional and longitudinal	ASA yes/no; NSAIDs yes/no	2300 dementia-free	Digits Forward and Backward portions of WAIS-R, CVLT, BVRT, TMT, Letter & Category Fluency, BNT, MMSE, BIMCT	SE	Cross-sectional: use of ASA related to better average performance across testing sessions on measures of verbal and visual learning, and memory and global mental status. Longitudinal: ASA related to greater prospective decline on BIMCT and the BVRT. Significant effects of ASA use were noted for the BVRT, the CVLT learning slope and short free recall, and the MMSE, and indicated better average levels of function for ASA users

**Clinical population**							

Ketterer, 1996; Coronary angiography	Regular ASA prophylactic therapy for IHD associated with emotional distress		ASA 80 to 325 mg	174 men	CMS, Framingham Type A Scale, KSSFC		ASA associated with less depression and anxiety or worry on the KSSFC

Stanford, 1999; HF or previouS OHT or CB			Usual schedule of drug therapy maintained	135 participants	Profile of Mood States	Mean and SEM	More positive mood in ASA groups due to less fatigue. Tension and TMD in ASA patients just failed to reach criterion for statistical significance

Broe; 2000; SOP Study dementia		Case-control	80% on ASA 175 mg; no high dose	163 NII >75 years old with different dementias categories, and 373 control subjects	NINCDS-ADRDA criteria for AD		Inverse association between ASA and AD, but not other dementia, not dosage-related

Mendlewicz, 2006; treatment-resistant DP	Accelerating effect of ASA in combination with fluoxetine	Open-label, uncontrolled	Treated openly during 4 weeks with ASA 160 mg/day in addition to their current antidepressant treatment	21 participants underwent >4 weeks SSRI	HDRS	Mean and SD	SSRI + ASA showed a global response rate of 52%. Remission was achieved in 43% of the total sample and 82% of the responder sample. In the responder group, a significant improvement was seen within week 1, which was sustained until day 28

Stolk, 2010; bipolar disorder	NSAIDs and glucocorticoids ameliorate bipolar symptoms		ASA 30 or 80 mg/day or ASA >80 mg/kg or non-selective NSAIDs or COX-2i or GCs + lithium	5145 participants receiving lithium	Incidence density of medication events (change in medication or increase of >30% of the current dose)	Incidence density ratio	Subjects receiving ASA 30 or 80 mg/day were 17% less likely to have a medication event; with >80 mg/kg ASA, non-selective NSAIDs, COX-2i, and GCs were not significant, but non- selective NSAIDs and GCs significantly increased medication events

3MSE, Modified Mini-Mental State Examination; AD, Alzheimer's disease; ADDTC, Alzheimer Disease Diagnostic and Treatment Centers; apoE, apolipoprotein E; ASA, acetylsalicylic acid (aspirin); AVLT, Auditory-Verbal Learning Test; BIMCT, Blessed Information-Memory-Concentration Test; BNT, Boston Naming Test; BVRT, Benton Visual Retention Test; CESDS, Center for Epidemiologic Studies Depression Scale; CIE, Canberra Interview for the Elderly; CMS, Cook-Medley Scale; COWAT, Controlled Oral Word Association Test; COX-2i, cyclooxygenase-2 inhibitor; CVF, Competing Values Framework; CVLT, California Verbal Learning Test; DSM-III-R, Diagnostic and Statistical Manual of Mental Disorders, 3rd revision; EBMT, East Boston Memory Test; F-U, follow-up; GC, Glucocorticoids; GDS, Geriatric Depression Scale; HDRS, Hamilton Depression Rating Scale; HR, hazard ratio; IHD, ischemic heart disease; JLOT, Judgment of Line Orientation; KSSFC, Ketterer Stress Symptom Frequency Checklist; MDD, Major Depressive Disorder; MMSE, Mini-Mental State Examination; MRI, magnetic resonance imaging; NART, National Adult Reading Test; NII, Non-institutionalized individuals; NINCDS-ADRDA, National Institute of Neurological and Communicative Diseases and Stroke/Alzheimer's Disease and Related Disorders Association; NINDS/AIREN, National Institute of Neurological Disorders and Stroke and Association Internationale pour la Recherché et l'Enseignement en Neurosciences; NSAID non-steroidal anti-inflammatory drug; OR, odds ratio; PFAQ, Pfeffer Activities Questionnaire; PMS, Profile of Mood States; SCID I RV-NP, Structured Clinical Interview for DSM-IV Axis I Disorders, Research Version, Non-patient; SD, standard deviation; SE, standard error; SEM, standard error of the mean; SLMT, Symbol Letter Modalities Test; SPMSQ, Short Portable Mental Status Questionnaire; SSRI, selective serotonin reuptake inhibitor; TMD, Total Mood Disturbance Score; TMT, Trail Making Test; VaD, vascular dementia; vs, versus; WAIS-R, (Wechsler Adult Intelligence Scale - Revised; WFT, Word Fluency Test.

**Table 2:** Summary of key randomized controlled trials evaluating the effects of aspirin use on mental illness and symptoms.

Author, year; study	Study design	Agent/dosage	Sample	Psych measure	Presentation of results	Key finding
**Non-clinical population**						

Dinnerstein, 1970	DB-RCT	ASA 600 mg + placebo 'energizer'/'tranquilizer' versus lactose 600 mg + placebo 'energizer'/'tranquilizer'	80 healthy male college students	CMS		ASA had no direct and fixed effect on mood, but acts to modulate the effect of placebo or other contextual variables

Lieberman, 1987	DB-RCT	2* to 6*sessions: caffeine 64 mg, or ASA 800 mg, or caffeine 64 mg + ASA 800 mg, or caffeine 128 mg + ASA 800 mg, or placebo in Latin-square design	20 healthy men 18 to 35 years old, caffeine intake <400 mg/day, non-smokers	6 sessions PMS, visual analog mood scales, NVMS, SSS, and performance tests	Mean and SEM	Caffeine alone and caffeine +ASA improved vigilance, self-reported efficiency and mood compared with ASA alone and placebo

Kang, 2007; Women's Health Study	Cohort study within a DB-RCT	ASA 100 mg or placebo on alternate days	6,377 women >65 years old	TICS-M, immediate and delayed recalls of the EBMT and delayed recall of the TICS-M, 10-word list and category fluency (naming as many animals as possible in 1 minute)	Mean and SD	ASA users did not differ in overall performance in any of the cognitive assessments, from the 1* assessment (after 5.6 years) to the 3* (after a mean 9.6 years), or in their average cognitive decline during 3 to 6 years of follow-up. ASA users performed better in category fluency, particularly in the final assessment

Kudielka, 2007	DB-RCT	ASA 100 mg, or propranolol 80 mg, or ASA 100 mg + propranolol 80 mg, or placebo	73 healthy subjects	TSST. Cortisol from six saliva samples taken before and after the stress exposure	Mean and SD	5 days: groups did not differ in their cortisol responses

**Clinical population**						

Clarke, 2003; VITAL trial collaborative group; dementia or MCI	DB-RC; 4-week placebo-controlled run-in period before randomization + 12-week treatment	ASA 81 mg, or placebo AND folic acid 2 mg + vitamin B12 1 mg, or placebo AND vitamin E 500 mg + vitamin C 200 mg, or placebo in 2 × 2 × 2 factorial design	149 NII, 12 to 26 patients MMSE score or <27 patients TICS-M score, naïve to study medications. Follow-up: 137 for biochemical outcomes, 128 for cognitive outcomes	TICS-M, MMSE, ADAS	Median percentage reduction	12 weeks of ASA was effective in reducing biochemical factors (thromboxane) associated with cognitive impairment in people at risk of dementia. No effect of treatment on cognitive function

AD2000, 2008; Alzheimer's disease	OL-RCT	ASA 75 mg yes/no	310 NII	MMSE, behavioral symptoms	Mean and SD	At 3 -year follow-up: no differences in scores, significantly higher risk of bleeding

Price, 2008; asymptomatic atherosclerosis	DB-RCT	ASA 100 mg or placebo	3350 participants 50-75 years old	Summary cognitive score = tests of memory, executive function, non-verbal reasoning, mental flexibility, and information processing	Mean and SD	At 5-year follow-up: no differences

Gałecki, 2009; first depressive episode	OL-RCT	fluoxetine 20 mg, or fluoxetine 20 mg + ASA 150 mg	77 participants	HDRS		No differences in HDRS between fluoxetine group and fluoxetine + ASA group

Laan, 2010; schizophrenic spectrum disorders	DB-RCT	ASA 1000 mg/day or placebo adjuvant to antipsychotic + pantoprazole 40 mg/day	70 adults PANSS ≥60, 2 items ≥4, illness duration <10 years. 2-week placebo run-in period, and only those who achieved over 80% compliance were randomized	PANSS	Mean and SD	Adjuvant ASA reduced overall psychopathology and positive symptoms at 3 months. No significant results in other subscales. ASA had greater effect on overall psychopathology in individuals with more altered immune function. ASA significantly reduced overall psychopathology in individuals with the lowest Th1:Th2 ratios

ADAS, Alzheimer's Disease Assessment Scale; ASA, acetylsalicylic acid (aspirin); DB, double-blind; EBMT, East Boston Memory Test; HDRS, Hamilton Depression Rating Scale; MCI, mild cognitive impairment; MMSE, Mini-Mental State Examination; NII, Non-institutionalized individuals; NVMS, Nestle Visual Analog Mood Scale; OL, open-label; PANSS, Positive and Negative Syndrome Scale; PMS, Profile of Mood States RCT, randomized controlled trial; SD, standard deviation; SEM, standard error of the mean; SSS, Stanford Sleepiness Scale; Th, T-helper; TICS-M, Telephone Interview for Cognitive Status - Modified; TSST, Trier Social Stress Test.

## Role of aspirin in mood disorders; clinical data

There is some evidence suggesting beneficial effects for aspirin in mood disorders, through a shortened onset of action of antidepressants [130]. However, there are negative epidemiological data from a study of 5,556 older men, which showed no association between current aspirin use and depression, although the men who discontinued aspirin had a greater odds ratio for depression compared with those who had never used aspirin [131]. In a treatment-intervention study of 70 patients with depression, administration of aspirin together with fluoxetine conferred a greater reduction of OS parameters compared with fluoxetine monotherapy [132]. Another randomized controlled clinical trial is currently underway to investigate the potential benefits of aspirin as an adjunctive treatment in bipolar depression [133].

## Role of aspirin in schizophrenia: clinical data

A recent study showed that aspirin reduces the core symptoms of schizophrenia [134]. Although the study had a relatively small number of participants (n = 70), it did show a benefit of aspirin 1,000 mg compared with placebo over 3 months of treatment. Using the Positive and Negative Symptoms Scale (PANSS), improvements were seen on the total PANSS and the positive subscale. Given the overlapping biomarker data in mood and psychotic disorders, this is an intriguing lead.

## Role of aspirin in Alzheimer's disease: clinical data

A number of studies have investigated the potential role for aspirin in the treatment and prevention of AD, although at this stage, its therapeutic potential has not been proven. Epidemiological investigations showed that individuals who used high-dose aspirin exhibited lower rates of AD and improved maintenance of cognition than those who did not use the drug [135]. However, a recent systematic review and meta-analysis of controlled intervention studies failed to show an improvement in indices of cognitive decline, but did find increased bleeding rates in patients taking aspirin [136]. However, given the short-term nature of many controlled studies, it is difficult for such studies to identify the risks and benefits of aspirin for primary prevention of AD in the longer term. Some authors have suggested that, in combination with docosahexaenoic acid, administration of low-dose aspirin may 'provide multiple levels of protection against the course of Alzheimer's' [137].

## Conclusion

The evidence presented in this review provides a window into a potentially new line of therapeutic targets for treating psychiatric and neurodegenerative disorders. Inflammation and OS seem to be active pathways not only in the disorders highlighted in this paper, but in other diverse medical comorbidites. Increased inflammation and OS seem to be underlying factors for each of the seemingly diverse conditions discussed in the review, and therefore, decreasing inflammation or inflammatory responses may have the potential to reduce risk and/or have therapeutic value in any or some of these disorders. Only trials will clarify this uncertainty. We are profoundly limited by phenomenologically based classifications in psychiatry, as nowhere else in medicine does phenomenology linearly reflect the underlying pathology, nor should we expect it to do so in psychiatry and related neurosciences. It is therefore unclear whether each disorder has unique patterns of inflammatory dysregulation that determine clinical symptoms, or whether these pathways are non-specific features, like pyrexia, of diverse underlying primary pathologies. This remains to be clarified. In this regard, biomarkers hold the promise of predicting therapeutic response, as there are tantalizing indications from a recent clinical trial of the TNF antagonist infliximab that treatment with anti-inflammatory agents may be beneficial only in patients with raised levels of baseline inflammatory markers [138]. Similarly, a recent cross-sectional study of 3687 men aged 69-87 years showed that older men with high homocysteine levels had a greater risk of depression, while those men who had raised homocysteine levels and were users of aspirin had a lower risk of depression [139].

There has been extensive characterization through basic science of the mechanisms by which aspirin might specifically reduce inflammation and OS and how this might subsequently lead to benefit in the clinical setting. Indeed, although there is a paucity of clinical trials investigating aspirin, the data provided thus far are promising. Randomized controlled trials of aspirin that include the analysis of biomarkers may not only provide further support for the use of aspirin, but may also serve to further characterize the underlying pathophysiology of these disorders. Further research is required to fully elucidate whether aspirin has clinical potential as an adjunctive treatment in psychiatry and neurology.

## Abbreviations

ATLs: aspirin-triggered lipoxins; C4: complement component 4; CCL2: chemokine (C-C motif) ligand 2; CDC42: cell division control protein 42 homolog; COX: cyclo-oxygenase; CRP: C-reactive protein; EGR3: Early growth response protein 3; GSH-Px: glutathione peroxidase; ICAM-1: intercellular adhesion molecule 1; IL: interleukin; iNOS: Inducible nitric oxide synthase; LPS: lipopolysaccharide; LXs: lipoxins; MDA: Malondialdehyde; MDD: major depressive disorder; MDEs: major depressive episodes; MPS: mononuclear phagocyte system; MS: multiple sclerosis; NF-κB: Nuclear factor-κB, O&NS, oxidative and nitrosative stress; OPG: osteoprotegerin; OS: oxidative stress; PAI-1: plasminogen activator inhibitor-1; PANSS: Positive and Negative Symptoms Scale; PD: Parkinson's disease; PET: positron emission tomography; PGE2: prostaglandin E2, PTX3, pentraxin-related protein; QUIN: quinolinic acid; sACC: subgenual anterior cingulate cortex; SOD: superoxide dismutase; TNF: tumor necrosis factor.

## Competing interests

MB has received grant/research support from the NIH, Cooperative Research Centre, Simons Autism Foundation, Cancer Council of Victoria, Stanley Medical Research Foundation, MBF, NHMRC, Beyond Blue, Geelong Medical Research Foundation, Bristol Myers Squibb, Eli Lilly, Glaxo SmithKline, Organon, Novartis, Mayne Pharma and Servier; has been a speaker for Astra Zeneca, Bristol Myers Squibb, Eli Lilly, Glaxo SmithKline, Janssen Cilag, Lundbeck, Merck, Pfizer, Sanofi Synthelabo, Servier, Solvay and Wyeth; and served as a consultant to Astra Zeneca, Bristol Myers Squibb, Eli Lilly, Glaxo SmithKline, Janssen Cilag, Lundbeck and Servier. OMD has received grant support from the Brain and Behavior Foundation, Simons Autism Foundation, Stanley Medical Research Institute, Lilly, NHMRC and ASBD/Servier. SM, HAD, CGD, JJM, MM, and AO declare no conflicts of interest.

## Authors' contributions

MB was responsible for the initial conception and drafting of the manuscript, and collating the contributions from the other authors. OD was responsible for contributing to sections pertaining to the biochemistry of aspirin, and also to the psychiatric clinical sections. HD was responsible for contributing to the mechanistic sections of the review. JMN and AON were responsible for contributing to the mechanistic sections, and also to the neurodegenerative clinical sections. SM and CD contributed to the biochemical and the clinical psychiatric sections. MM contributed to the underlying-pathways sections, and assisted in bringing together all the theoretical components. All authors have read and approved the final manuscript.

## Pre-publication history

The pre-publication history for this paper can be accessed here:

http://www.biomedcentral.com/1741-7015/11/74/prepub
